# Characteristics of Primary and acquired multidrug-resistant tuberculosis patients in West Java, Indonesia

**DOI:** 10.1016/j.ijregi.2026.100960

**Published:** 2026-07-18

**Authors:** Prayudi Santoso, Alya Putri Khairani, Eppy Buchori, Lidya Chaidir, Arto Yuwono Soeroto

**Affiliations:** 1 Pulmonology and Critical Care Medicine Division, Department of Internal Medicine, Faculty of Medicine, Hasan Sadikin General Hospital, Universitas Padjadjaran, Bandung, Indonesia; 2 Department of Internal Medicine, Faculty of Medicine, Hasan Sadikin General Hospital, Universitas Padjadjaran, Bandung, Indonesia; 3 Department of Radiology, Faculty of Medicine, Hasan Sadikin General Hospital, Universitas Padjadjaran, Bandung, Indonesia; 4 Department of Biomedical Sciences, Faculty of Medicine, Hasan Sadikin General Hospital, Universitas Padjadjaran, Bandung, Indonesia; 5 Center for Translational Biomarker Research, Universitas Padjadjaran, Bandung, Indonesia

**Keywords:** Acquired MDR-TB, Indonesia, Primary MDR-TB, TB contact, Tuberculosis

## Abstract

**Objectives:**

The increasing incidence of primary multidrug-resistant tuberculosis (MDR-TB) creates a new burden in national tuberculosis (TB) control. Understanding the characteristics of primary MDR-TB aids in predicting its spread. This study aimed to determine the differences in characteristics of primary and acquired MDR-TB patients in a hospital setting in Indonesia.

**Methods:**

This is a retrospective cohort study of adult patients (aged >18 years) with pulmonary MDR-TB who received treatment at Hasan Sadikin General Hospital, West Java, Indonesia. Patients who finished MDR-TB treatment between 2020 and 2022 were included. Patients whose therapeutic outcomes could not be determined and those with incomplete data were excluded.

**Results:**

This study included 337 participants; 32% were primary MDR-TB and 68% were acquired MDR-TB. Findings showed a significant difference between primary and acquired MDR-TB by contact with a TB index case. Primary MDR-TB was 4.31 times higher in patients who had contact (prevalence odds ratio 4.31, 95% confidence interval 2.65-7.01). Other characteristics showed some differences but were not statistically significant.

**Conclusion:**

Contact with the TB index case was found to differ significantly between primary and acquired MDR-TB. The transmission-driven nature of drug-resistant TB underscores the urgent need to reposition contact investigation and preventive therapy as central pillars of the MDR-TB control strategy.

## Highlights

• Primary multidrug-resistant tuberculosis made up 32% of 337 cases.
• Tuberculosis contact raised primary multidrug-resistant tuberculosis odds four-fold.
• Age, body mass index, and clinical features did not differ significantly.
• Linezolid resistance was found in primary cases.
• Acquired multidrug-resistant tuberculosis showed more diverse drug resistance.

## Introduction

Resistance to anti-tuberculosis drugs is a significant constraint that is often encountered in tuberculosis (TB) control and creates an additional burden on TB control programs [1]. Multidrug-resistant tuberculosis (MDR-TB) is TB caused by *Mycobacterium tuberculosis* complex (*M tuberculosis*) that is resistant to isoniazid (H) and rifampicin (R) simultaneously, with or without resistance to other first-line anti-TB drugs [2]. Globally, the estimated proportion of people with TB who had multidrug-resistant or rifampicin-resistant TB was 3.2% among new cases (primary resistance cases) and 16% among those previously treated (acquired resistance cases) [1]. These figures may be underestimated globally because utilization of rapid molecular testing was lower in primary case TB patients compared with those in acquired cases (59% vs 81%, respectively) [3]. The utilization of rapid molecular testing in Indonesia as the gold standard diagnostic tool of newly diagnosed TB cases only reached 48.8% of the national target of 65%, leading to an underestimated proportion of newly diagnosed drug-resistant TB cases, mainly primary MDR-TB cases [4].

Several studies have indicated that primary MDR-TB exhibits different characteristics compared with acquired MDR-TB. In China, variations were found in age, sex, smoking habits, and sputum resistance patterns. In Ethiopia, differences were noted in the history of TB contact, nutritional status, and sputum conversion time. Research in India highlighted differences in the type and severity of lung lesions seen on chest radiographs. In South America, the prevalence of comorbidities such as diabetes mellitus (DM) and human immunodeficiency virus (HIV) also varied. Additionally, treatment outcomes differed between primary and acquired MDR-TB cases [[5], [6], [7], [8], [9], [10], [11], [12]]. However, most MDR-TB studies in Indonesia have characterized patient populations without explicitly stratifying cases by their resistance acquisition pathway. This gap limits the evidence base available to guide the targeting of prevention and control interventions.

Understanding the epidemiological and clinical characteristics that distinguish primary from acquired MDR-TB in the Indonesian context is a foundational prerequisite for designing TB control strategies that are appropriately matched to the local drivers of drug resistance. This study aimed to determine the differences in characteristics of primary and acquired MDR-TB patients in a hospital setting in Indonesia.

## Methods

### Study setting and population

This retrospective cohort study was conducted at Hasan Sadikin General Hospital, West Java, Indonesia. Data were collected from medical records and the Tuberculosis Information System (SITB) of the Indonesian Ministry of Health. Data were first accessed for research purposes on February 13, 2023. Each patient's demographic and clinical information, microbiologic and radiographic results, treatment plans, and outcomes were documented. Treatment history data obtained from medical records and the SITB registry were cross-verified with the primary health care center (Puskesmas) in each patient's area of residence. This approach was feasible because sputum smear microscopy performed at private health care facilities in Indonesia is routinely referred to the local Puskesmas for processing and recording, establishing the Puskesmas as the lowest-level community repository of TB diagnostic and treatment data. This cross-verification was applied systematically to all enrolled patients to capture prior treatment episodes that may not have been recorded in hospital-based records or the national registry.

The study population consisted of adult pulmonary MDR-TB patients (aged >18 years) who received MDR-TB treatment from July 2020 to September 2022 and whose treatment had been discontinued due to completion or other reasons (treatment failure, loss to-follow-up, or death). Patients who transferred to other health care facilities with undetermined treatment results and cases with missing data were excluded. All patients were known to be resistant to rifampicin through GeneXpert and resistant to other drugs through drug susceptibility testing or line probe assay. Baseline chest radiographs were reevaluated by two radiologists. Patient data were reidentified and analyzed anonymously.

## Independent variables and definitions

This study analyzed 12 independent variables: age, sex, body mass index (BMI), smoking habits, HIV status, DM, contact with TB patient, sputum conversion time, consolidation in baseline chest radiograph, severity of lung lesions, sputum culture resistance pattern, and treatment outcome. Only patients who had all of the values were selected.

For the statistical analysis conducted below, primary MDR-TB refers to resistance to rifampicin and isoniazid, found in *M tuberculosis* strains taken from patients who have never undergone treatment, while acquired MDR-TB refers to resistance found in strains isolated from patients who have previously received TB treatment [13]. Patients’ self-reports of previous TB treatment were cross-referenced with SITB records to avoid bias. Sputum culture resistance pattern and treatment outcomes were defined using the World Health Organization (WHO) definitions in 2020 [2]. Sputum conversion time refers to the interval from treatment initiation to the first of two consecutive negative cultures [2]. The definition of contact with a TB patient was also based on the WHO definition, in which an individual who self-reported living in the same confined space as the TB patient (either overnight or frequently spending extended hours during the day) within the 3 months before the beginning of TB treatment [2]. The definition of severity of lung lesions parallels that of the National Tuberculosis and Respiratory Disease Association, where mild lesions contained non-cavitary opacities of low-to-moderate density, limited in total volume to the lung area above the second costal cartilage and the T4/T5 vertebrae; moderate lesions showed bilateral or unilateral involvement restricted to one full lung volume of moderate density, one-third lung volume if dense/confluent, or a total cavity diameter under 4 cm; lastly, severe lesion involvement exceeded the criteria for moderate lesions [14]. Independent evaluations were carried out by two radiologists blinded to the clinical data.

### Statistical analysis

Demographic and characteristic data of primary and acquired MDR-TB patients were described descriptively in tables. The results were then analyzed using unpaired bivariate analysis, including the χ² test or Fisher exact test for categorical variables and the Mann-Whitney *U* test for numerical data. The initial sample size calculation using the Fleiss formula for comparing two proportions without the continuity correction factor, assuming a 95% confidence level and margin of error of 0.05, was 208 samples [15]. Data analysis was conducted using SPSS version 23 with a 95% confidence interval (CI) and *P* < 0.05 considered statistically significant.

## Results

**Figure 1:**
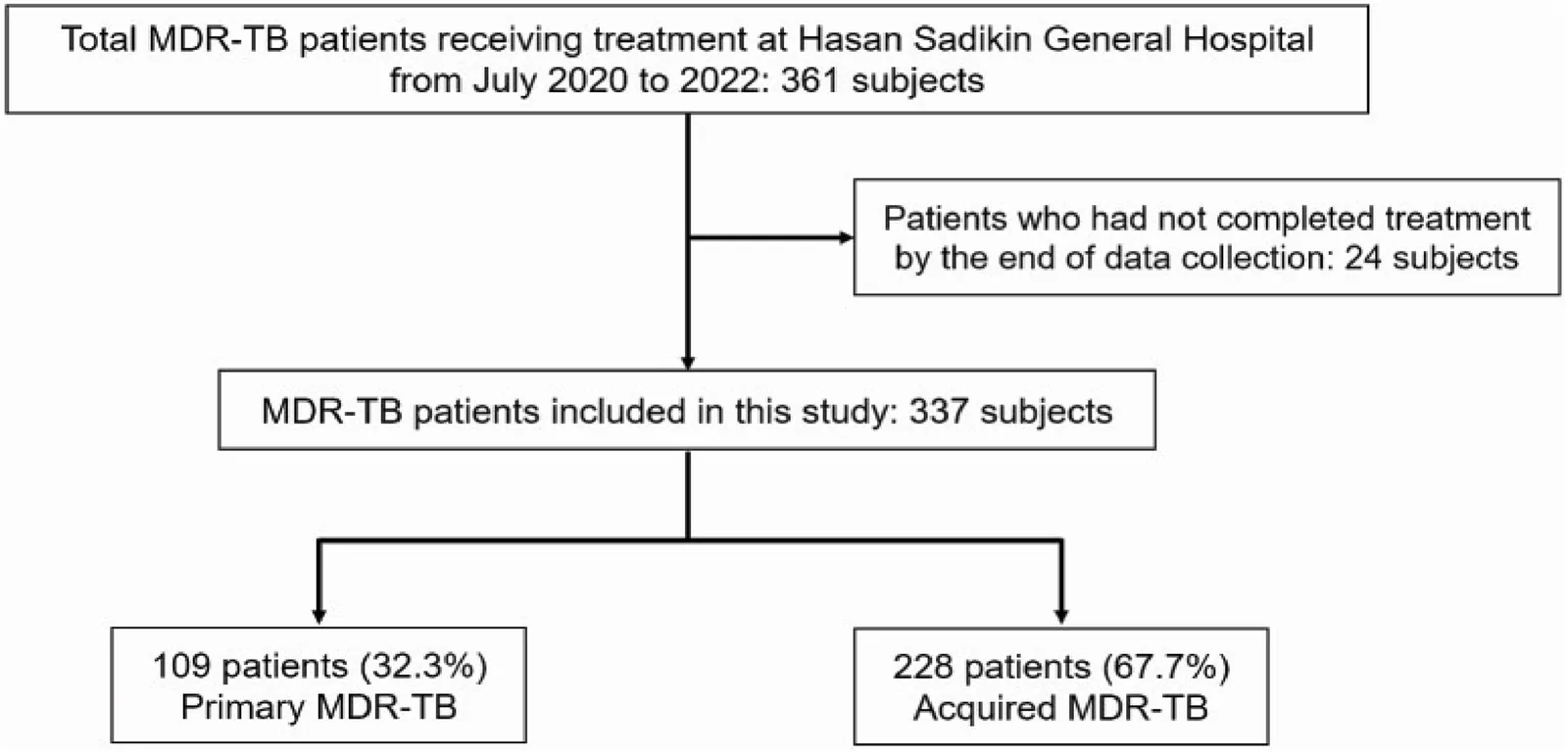
Flowchart of study participants. MDR-TB, multidrug-resistant tuberculosis; TB, tuberculosis.

The Figure 1 shows 361 patients with MDR-TB undergoing treatment at the MDR-TB Clinic of Hasan Sadikin General Hospital from July 2020 to September 2022. A total of 337 patients met the inclusion and exclusion criteria for the study. Out of patients receiving treatment, 32% were primary MDR-TB, while the remaining 68% were acquired MDR-TB.

The baseline characteristics are summarized in Table 1. Both primary and acquired MDR-TB predominantly fell within the age range of 25 to 44 years. Overall, the difference in age distribution between primary and acquired MDR-TB was not statistically significant (*P* = 0.256). Within this overall pattern, the youngest age group (aged 18-24) and the oldest age group (aged ≥ 65) both showed a higher proportion of primary MDR-TB (22.9% and 6.5%, respectively) compared with acquired MDR-TB (14.1% and 4.8%, respectively), whereas the 25 to 44 and 45 to 64 age groups showed a higher proportion of acquired MDR-TB. The distribution of sex was not found to be significantly different between the two MDR-TB groups. Male predominance was observed in both cases. The proportion of smokers was higher among primary MDR-TB patients compared with acquired MDR-TB patients. The proportion of patients who had contact with a TB patient was higher in primary MDR-TB compared with acquired MDR-TB. The study revealed that individuals who had contact with a TB patient had a 4.31 times higher odds of developing primary MDR-TB compared with those who did not.

**Table 1:** Demographic and clinical characteristics of primary and acquired MDR-TB.

Characteristic	Total (*n*)	MDR-TB		*P*	POR (95% CI)
		Primary n (%)^a^	Acquired n (%)^a^		
Demographic characteristics					
Age (years), median (IQR)	337	38 (56)^a^	39 (63)^a^	0.256^b^	-
Age group (years)				0.183^c^	
18-24	57	25 (22.9)	32 (14.1)		
25-44	156	46 (42.2)	110 (48.2)		
45-64	106	31 (28.4)	75 (32.9)		
≥65	18	7 (6.5)	11 (4.8)		
Sex				0.281^c^	-
Men	193	67 (61.5)	126 (55.3)		
Smoking habit				0.363^c^	-
Yes	158	55 (50.5)	103 (45.2)		
Contact with TB patient				<0.001^c^^,^^d^	4.31 (2.65-7.01)
Yes	118	63 (57.8)	55 (24.1)		
Clinical characteristics					
BMI (kg/m^2^)				0.14^c^	-
Median (IQR)		18.07 (16)^a^	17.65 (22.5)^a^		
Underweight	198	60 (55.0)	138 (60.6)		
Normal	104	36 (33.0)	68 (29.8)		
Overweight	22	11 (10.2)	11 (4.8)		
Obese	13	2 (1.8)	11 (4.8)		
HIV status				0.733^e^	-
Yes	10	4 (3.7)	6 (2.6)		
Diabetes mellitus				0.672^c^	-
Yes	105	35 (32.1)	70 (30.7)		
Sputum conversion time				0.083^c^	-
≤ 2 months	150	49 (45.0)	101 (44.3)		
> 2 months	21	8 (7.3)	13 (5.7)		
No conversion occurred	42	10 (9.2)	32 (14.0)		
Negative culture	16	1 (0.9)	15 (6.6)		
Indeterminate	108	41 (37.6)	67 (29.4)		
Consolidation				0.717^c^	-
Unilateral	90	29 (26.6)	61 (26.8)		
Bilateral	223	74 (67.9)	149 (65.4)		
No consolidation	24	6 (5.5)	18 (7.8)		
Cavity					
Detected	130	36 (33.0)	94 (41.2)	0.156^c^	-
Diameter < 4 cm	107	28 (77.7)	79 (84.04)	0.402^c^	-
Diameter ≥ 4 cm	23	8 (23.3)	15 (16.96)		
Severity lung lesion				0.224^c^	-
Normal	2	2 (1.8)	0 (0)		
Mild	97	32 (29.4)	65 (28.5)		
Moderate	187	58 (53.2)	129 (56.6)		
Severe	51	17 (15.6)	34 (14.9)		
Outcome				0.196^c^	-
Favorable	150	43 (39.4)	107 (46.9)		
Unfavorable Failed Death Lost to follow-up	187	66 (60.6) 17 (15.6) 19 (17.5) 30 (27.5)	121 (53.1) 36 (15.8) 46 (20.2) 39 (17.1)		

Abbreviations: BMI, body mass index; CI, confidence interval; HIV, human immunodeficiency virus; IQR, interquartile range; MDR-TB, multidrug-resistant tuberculosis; POR, prevalence odds ratio; TB, tuberculosis.

a Unless otherwise specified.

b *P* values calculated using the Mann-Whitney U test,

c *P* values calculated using the χ² test, or

d Significant at *P* < 0.05.

e *P* values calculated using the Fisher exact test.

Table 1 also shows clinical characteristics of the patient in this study. Overall, the difference in BMI distribution between primary and acquired MDR-TB was not statistically significant (*P* = 0.141). The proportion of underweight individuals was lower in primary MDR-TB compared with acquired MDR-TB (55.0% vs 60.6%), whereas the prevalence of overweight individuals was higher in primary MDR-TB compared with acquired MDR-TB (10.2% vs 4.8%), whereas the proportion of obese individuals was, conversely, higher in acquired MDR-TB compared with primary MDR-TB (4.8% vs 1.8%). The prevalence of HIV coinfection did not show a statistically significant difference between the 2 instances of MDR-TB (*P* = 0.733). The prevalence of DM was seen to be greater in primary MDR-TB patients compared with acquired MDR-TB patients, although this difference was not statistically significant (*P* = 0.672).

The difference of proportion in sputum conversion within 2 months between both cases of MDR-TB was found to be not statistically significant (*P* = 0.083). However, the percentage of patients undergoing sputum conversion was somewhat higher in primary MDR-TB compared with acquired MDR-TB. Acquired MDR-TB exhibited a higher prevalence of remaining positive sputum culture at the end of treatment. Lung consolidation in baseline chest radiograph was observed in both primary and acquired MDR-TB, with a proportionate distribution (*P* = 0.717). Additionally, the severity of lung lesions in both cases with MDR-TB was also shown to be similar (*P* = 0.224). Therefore, there were no significant differences in the size of lung cavities detected (*P* = 0.156) or in cavity diameter (*P* = 0.292), lung consolidations, and severity of lung lesions between primary and acquired MDR-TB.

Cases of pre–extensively drug-resistant TB (pre-XDR-TB) and extensively drug-resistant TB (XDR-TB) were found in primary cases. Although the overall difference in treatment outcome distribution between primary and acquired MDR-TB was not statistically significant (*P* = 0.196), primary MDR-TB showed a numerically higher proportion of unfavorable outcomes, driven mainly by a higher proportion of patients lost to follow-up. Based on Table 2, the resistance patterns in acquired MDR-TB were observed to exhibit more diversity, although the overall difference in resistance pattern distribution was not statistically significant (*P* = 0.653). Both MDR-TB groups showed notable aminoglycoside resistance. Additionally, one case each of primary MDR-TB and primary pre-XDR-TB with resistance to linezolid was identified.

**Table 2:** Distribution of drug resistance among primary and acquired MDR-TB.

Characteristic	Total (n)	MDR-TB		*P*	POR (95% CI)
		Primary n (%)	Acquired n (%)		
Resistance pattern				0.653^a^	-
MDR	263	86 (78.9)	177 (77.6)		
H-R		82 (75.2)	167 (73.2)		
H-R-Z		3 (2.8)	8 (3.5)		
H-R-Cfz		-	2 (0.9)		
H-R-Lzd		1 (0.9)	-		
Pre-XDR	59	19 (17.4)	40 (17.5)		
H-R-Mfx		2 (1.8)	4 (1.8)		
H-R-Lfx		-	1 (0.4)		
H-R-Ofx		-	4 (1.8)		
H-R-Lfx-Mfx		3 (2.8)	7 (3.3)		
H-R-Lfx-Mfx-Z		2 (1.8)	1 (0.4)		
H-R-Lfx-Mfx-Ofx		-	3 (1.3)		
H-R-Lfx-Mfx-Am-Cfz-Z		-	1 (0.4)		
H-R-Mfx-Ofx-Km-Cm		-	1 (0.4)		
H-R-Km		-	3 (1.3)		
H-R-Cm		-	1 (0.4)		
H-R-Km-Cm		-	1 (0.4)		
H-R-Km-Cm-Am		11 (10.1)	11 (4.8)		
H-R-Z-Cm		-	1 (0.4)		
H-R-Z-Am-Lzd		1 (0.9)	-		
H-R-E-Km-Cm-Am		-	1 (0.4)		
XDR	15	4 (3.7)	11 (4.9)		
H-R-Lfx-Mfx-Km-Am-Cm		1 (0.9)	5 (2.2)		
H-R-Lfx-Mfx-Ofx-Km-Am-Cm		1 (0.9)	1 (0.5)		
H-R-Lfx-Mfx-Ofx-Km-Cm		-	2 (0.7)		
H-R-Lfx-Km-Am-Cm		2 (1.9)	1 (0.5)		
H-R-Lfx-Mfx-Ofx-Gfx-Cm-Eto-Lzd		-	1 (0.5)		
H-R-Lfx-Mfx-Km-Am-Cm-Z		-	1 (0.5)		

Abbreviations: Am, amikacin; Cfz, clofazimine; CI, confidence interval; Cm, capreomycin; E, ethambutol; Gfx, gatifloxacin; H, isoniazid; Km, kanamycin; Lfx, levofloxacin; Lzd, linezolid; MDR-TB, multidrug-resistant tuberculosis; Mfx, moxifloxacin; Ofx, ofloxacin; POR, prevalence odds ratio; pre-XDR, pre–extensively drug-resistant; R, rifampicin; TB, tuberculosis; XDR, extensively drug-resistant; Z, pyrazinamide.

*P* values calculated using the (^a^) χ² test.

## Discussion

Research on primary MDR-TB in Indonesia is scarce, particularly when compared with the features of acquired MDR-TB. This study was the first conducted at the MDR-TB Clinic of Hasan Sadikin General Hospital focusing on primary MDR-TB. The study revealed a significant difference in terms of contacts with TB patients between primary and acquired MDR-TB cases. Individuals who had contact with a TB patient were 4.31 times more likely to have primary MDR-TB compared with those who did not have contact (prevalence odds ratio 4.31, 95% CI 2.65-7.01, *P* < 0.001). The results correspond with the previous study by Bayissa et al. [8], which demonstrated that contact with a TB or MDR-TB patient significantly increases the probability of transmitting resistant *M tuberculosis* (57.1%; *P* = 0.004 [adjusted odds ratio 4.15, 95% CI 1.75-9.84]). The robust correlation identified between prior TB contact and primary MDR-TB underscores critical programmatic implications for public health strategies. Differentiating between primary and acquired drug resistance is vital for tailoring epidemiological interventions where primary resistance indicates a failure to interrupt ongoing transmission, demanding enhanced containment strategies [16]. Consequently, distinguishing between transmission-driven and treatment-driven MDR-TB is essential for the strategic prioritization and allocation of public health resources.

A single individual infected with MDR-TB has the potential to transmit the disease to 22 other individuals, leading to the development of more than two new cases of primary MDR-TB [5,17]. The finding that TB contact serves as a dominant predictor of primary MDR-TB reinforces the imperative for systematic contact investigations among close contacts of confirmed cases. However, active contact tracing remains one of the least executed components within the national TB control program [18]. Another investigation of *M tuberculosis* transmission hotspots in index cases showed that primary MDR-TB patients are superspreaders [19]. Optimizing active tuberculosis detection and treatment is insufficient for elimination efforts; eradicating MDR-TB as a public health threat necessitates the identification and treatment prior to disease progression. Consequently, contact investigations for MDR-TB cases must be prioritized to facilitate both secondary active case-finding and the timely administration of TB preventive therapy [20]. Evidence indicates that TB preventive therapy is both safe and effective for individuals exposed to drug-resistant TB. A 6-month course of levofloxacin has been shown to lower the odds of developing TB by 62% within a year among household contacts of index cases with multidrug-resistant or rifampicin-resistant TB [21].

The proportion of primary MDR-TB in this study reached 32.3%, with 67.7% for acquired MDR-TB. These proportions are higher than the findings in Chongqing, China, which reported 24.5% as primary cases and 75.5% as acquired cases [5]. This disparity may be associated with the difference in transmission intensity and population density in Indonesia. The higher prevalence of primary MDR-TB in West Java likely reflects a robust local transmission chain rather than elevated treatment-related selective pressure [22].

The proportion of younger individuals (aged 18-24) was observed to be higher in primary MDR-TB, whereas the proportion in older age groups (aged 45-64) was higher in acquired MDR-TB; notably, the oldest group (aged ≥65) again showed a higher proportion in primary MDR-TB. The higher proportion of acquired MDR-TB in the working-age population, who are frequently obligated to carry out outdoor tasks, which heightens the likelihood of transmitting *M tuberculosis* with resistance. Resistance to TB treatment arises in persons who have undergone previous treatment for TB. This resistance is more likely to occur in older patients. Similar findings were also reported in other research centers [5,23].

The study predominantly comprised male patients with MDR-TB, consistent with previous research [5,23]. Men have higher odds of being exposed to odds related to social interactions. Moreover, women exhibit a more robust immune response to antigenic stimuli in comparison to men due to differences in sex hormones [6]. Primary MDR-TB patients had a higher proportion of smokers compared with acquired cases, as revealed in this study. Smoking habits in index cases increase new infections by more than 10% due to transmission in TB cases, hence aligning with the higher proportion of smokers in primary MDR-TB cases [24].

In this study, the overall difference in BMI distribution between MDR-TB patients was not statistically significant but the nutritional status was found to differ between MDR-TB patients. The proportion of underweight individuals was higher in acquired MDR-TB, and the proportion of normal-to-overweight individuals was higher in primary MDR-TB, whereas the proportion of obese individuals was, conversely, higher in acquired MDR-TB (a pattern that does not follow a simple linear trend and may reflect the small number of patients within the obese subgroup). The predominant pattern nonetheless is consistent with previous studies. The higher prevalence of underweight status in acquired MDR-TB patients likely reflects the cumulative catabolic burden of prolonged, recurrent infection. Conversely, the predominance of normal-to-overweight profiles in primary MDR-TB cases indicates recent, exogenous transmission into a host prior to the onset of chronic, disease-induced metabolic deterioration [8,25].

There was no notable distinction found between the MDR-TB patients coinfected with HIV. Another meta-analysis revealed contrary findings, indicating that HIV infection was linked to primary MDR-TB but not to acquired MDR-TB [12]. The lack of a significant between-group difference in HIV coinfection is likely attributable to the low background prevalence of HIV in West Java, which statistically limits the capacity to detect differential associations [26]. Results showed that the number of primary MDR-TB patients with DM was higher than in acquired MDR-TB patients. This result is consistent with previous meta-analyses [27]. Ruesen et al. [28] proposed that the higher mutation rate of resistance among TB-DM patients cannot be explained solely by a history of previous TB treatment, as the association between diabetes and resistance is also evident in patients with new MDR-TB cases.

There was no noteworthy distinction in terms of lung consolidation and cavities between the MDR-TB cases. This result is consistent with the previous study by Rai and Alok [9]. The severity of lung lesions on baseline chest radiograph was projected differently between primary and acquired MDR-TB [9,10]. However, this study found that the proportion of severity in primary MDR-TB did not exhibit any significant differences compared with acquired MDR-TB. This can be affected by the time interval between the onset of symptoms and diagnosis. The study conducted by Song et al. [29] found that primary MDR-TB exhibited a significantly greater occurrence of nodular consolidation only when the case had a history of less than 1 month and/or more than 4 months (*P* < 0.001). The lack of radiological differentiation in this study does not imply that primary and acquired MDR-TB are pathophysiologically identical on imaging. Instead, this absence of variation reflects the confounding effects of heterogeneous diagnostic delays, the convergence of radiological patterns over time, and study-design limitations that precluded temporal standardization of imaging findings.

There was no significant difference observed in the pattern of sputum culture resistance between the two MDR-TB patient groups. However, the finding of linezolid resistance in primary resistance cases in this study is noteworthy. One study in MDR *M. tuberculosis* isolates has shown a correlation between increases in linezolid minimum inhibitory concentration and the use of fluoroquinolones and kanamycin. The induction of efflux pump expression after exposure to those drugs plays a role in initiating a process that leads to subsequent resistance caused by high-level mutations [30]. The detection of linezolid resistance in a primary MDR-TB case indicates an inadequate efficacy of MDR-TB transmission surveillance. Another notable finding is the presence of resistance to aminoglycosides in both MDR-TB cases.

There was no significant difference in the proportion of pre-XDR-TB and XDR-TB between the cases. However, the proportions of pre-XDR-TB and XDR-TB in primary resistance in this study were higher compared with previous studies [3,5,8]. The proportion of pre-XDR-TB and XDR-TB in this study emphasizes the importance of being highly aware of primary MDR-TB cases.

The sputum conversion time was found not to differ significantly between primary and acquired MDR-TB. The inability to determine the sputum conversion status may be related to a greater number of patients lost to follow-up. Lastly, the treatment outcome was found not to have significant differences between primary and acquired MDR-TB. Comparable treatment outcomes do not diminish the clinical importance of early primary MDR-TB diagnosis. Because success rates converge following prolonged second-line regimens, the principal utility of early detection lies in mitigating the upstream consequences of delayed diagnosis [22]. Prompt identification shortens both ineffective first-line therapy and the community transmission window, thereby facilitating immediate source isolation, effective second-line treatment, and rapid contact tracing (including WHO-recommended levofloxacin preventive therapy for household contacts).

Limitations of this study include its single-center, medical record–based retrospective design and the inclusion of only patients with complete data, which may introduce selection bias and may not accurately reflect the diverse characteristics of primary MDR-TB. Moreover, the lack of molecular epidemiological confirmation precludes definitive differentiation between *de novo* transmission and unrecorded prior treatment exposure as the mechanism of resistance in treatment-naïve patients. Furthermore, although treatment history was cross-verified with local Puskesmas records, residual misclassification bias cannot be fully excluded, as some patients who received prior treatment exclusively through private providers without sputum smear referral may have been incorrectly classified as treatment-naïve. Additionally, the study did not assess the duration from the onset of MDR-TB symptoms to diagnosis, thus being unable to estimate conditions that might introduce bias in the results of chest radiographic findings or treatment outcomes. The reliance on routine clinical documentation rather than standardized, blinded radiological reassessment also introduces potential measurement bias and interobserver variability.

## Conclusion

The predominance of TB contact history as a leading odds factor for primary MDR-TB in this study confirms the transmission-driven nature of drug-resistant tuberculosis and underscores the urgent need to reposition contact investigation and preventive therapy as central pillars (not adjuncts) of the national MDR-TB control strategy. Consequently, these findings mandate that policymakers and health authorities strengthen local contact tracing infrastructure, integrate preventive regimens into routine management pathways, and implement district-level monitoring systems to track evaluation and completion rates.

## CRediT authorship contribution statement

**Prayudi Santoso:** Writing – review & editing, Writing – original draft, Methodology, Conceptualization. **Alya Putri Khairani:** Writing – review & editing, Formal analysis, Data curation. **Eppy Buchori:** Resources. **Lidya Chaidir:** Writing – review & editing, Supervision. **Arto Yuwono Soeroto:** Writing – review & editing, Supervision.

## Declaration of competing interest

The authors have no competing interests to declare.
